# Supplementary material for: The impact of Chinese adolescents visual art participation on self-efficacy: A serial mediating role of cognition and emotion
Source: PLoS One. 2023 Nov 22;18(11):e0288379. doi: 10.1371/journal.pone.0288379 (PMC10664882; doi:10.1371/journal.pone.0288379)
Supplement: Attachment — Submitted filename: PLOS ONE Manuscript Review-Chinese Adolescents Visual Arts.docx [file pone.0288379.s002.docx]

| **Reviewer’s comment** | |
| --- | --- |
| 1. | **Abstract**  **The abstract reads well and captures the essence of the study.** |
| 2. | **Introduction:** This section reads more like a review of literature than an introduction. There were far too many references to existing literature, indicating that this study was based on a thorough review of related literature. It is expected that the authors had a reason for conducting a literature review in the first place. That is what we are expecting to see in the introduction. That will be the impetus for the study. This could be conceptualised better without relying on too many references to related literature.  It is worth noting that, contrary to the authors' assertion, some extracurricular activities can be relatively inexpensive. That could be why children from low-income families excel in professional football, a sport that is considered extracurricular at the primary school level.  It might be useful to mention the range of skills that a child is likely to acquire at the primary/secondary school age, as well as how art can stimulate those skills.  I noticed multiple sources (citations) for each claim, indicating thorough research. However, you might want to limit it to three or four references. In-text citations abound on the first few pages. |
| 3. | **Literature Review:**  The array of references from related literature demonstrates the breadth of the research here. However, the literature review should be more critical. It is dense with references to other people's work, with little input from the authors on whether they agree or disagree with the positions in the existing literature. The references (works cited) must be contextualised within the Chinese environment - why do the authors agree with those positions? And why don't they agree based on local observation? Again, a portion of the literature review should have been included in the introduction section, and similar arguments were presented in both the introduction and the literature review section.  Secondly, the literature review section should be organised in the same order that the concepts were introduced: art participation, cognition, emotion, and self-efficacy. As a result, I would have expected a conceptual approach in the section on literature review.  There were a few spelling mistakes, sentence construction issues, and grammatical errors in this and the other sections. On the fourth page of the literature review section, there were a couple of incomplete sentences, such as "Likewise, given that primarily girls participate in extracurricular activities such as painting, drawing, sculpturing, crafting, and designing that require extensive creativity." |
| 4. | **Hypotheses:**  This is concise and clear. The structure of the literature review, on the other hand, should have followed the same order as the hypothesis presented. This will facilitate reading and improve comprehension. |
| 6. | **Materials and Methods:**  **This section is well-written and lends credibility to the researchers' expertise. But what about the research design? And why do you call this research "cross-sectional"?** |
| 7. | **Findings:**  A logical approach to presenting the findings in such a way that they align with the hypothesis would have facilitated reading and comprehension. To address this, I propose that the findings be restructured. |
| 8. | **Discussion**  I noticed that the six hypotheses were divided into two broad categories in the discussion section. While this is good, it may also be beneficial to inform the readers of the reasoning behind this decision.  Concerning the statement, "Ultimately, this paper discovers that, in addition to academic benefits, art education promotes nonacademic skills, which are the primary agents for academic performance," I find it difficult to associate nonacademic skills as an agent of academic performance. This statement should be clarified in the final paragraph of the discussion section. |
| 9. | **Conclusion:**  While the conclusion discusses the relationship between art cognition and emotion, it is silent on the relationship between participants' socioeconomic status and art participation. The conclusion should address the two broad areas of focus of this study in order to be complete.  On the last page of your ethical statement, double-check the spelling. The ethical statement, not the ethnic statement, should be used. |
