# Supplementary material for: The impact of Chinese adolescents visual art participation on self-efficacy: A serial mediating role of cognition and emotion
Source: PLoS One. 2023 Nov 22;18(11):e0288379. doi: 10.1371/journal.pone.0288379 (PMC10664882; doi:10.1371/journal.pone.0288379)
Supplement: Attachment — Submitted filename: Response to Reviewers Comments.docx [file pone.0288379.s003.docx]

Response to Reviewers' Comments

Dear Editors and reviewers,

I am writing this letter on behalf of my coauthor regarding our manuscript PONE-D-23-06961, entitled " ***The Impact of Chinese Adolescents Visual Art Participation on Self-Efficacy: A Serial Mediating Role of Cognition and Emotion***." We want to express our appreciation to the respected editors and reviewers for providing us the constructive comments and suggestions to shape our manuscript for quality publication. According to our original reviewer's last review, we have obtained critical and rational minor suggestions to complete the manuscript's structure and content before the official publication. As you can see below, we have responded to each comment given by the reviewer corresponding to each page's revision has been made.

**Reviewer 1 Comments**

**Comment 1:** The Research technically sound, & the data support the conclusions

The Statistical analysis performed appropriately and rigorously

The Authors made all data underlying the findings in their manuscript fully available

The Manuscript presented in an intelligible fashion and written in standard English, The way of writing very clear, correct, & unambiguous

**Response:** We genuinely appreciate the profound admiration for the work, which significantly benefits us.

**Reviewer 2 Comments**

**Comment 1**: The abstract reads well and captures the essence of the study.

**Response:** Thank you for the appreciation.

**Comment 2**: There were far too many references to existing literature, indicating that this study was based on a thorough review of related literature. It is expected that the authors had a reason for conducting a literature review in the first place.

# Response: Thank you for the reminder; we made sure that the number of citations is managed to keep the concept flow.

**Comment 3:** It is worth noting that, contrary to the authors' assertion, some extracurricular activities can be relatively inexpensive.

**Response:** We considered the suggestion. Unlike inexpensive extracurricular activities, the literature addressed the role of visual art in developing children's cognitive and non-cognitive development.

**Comment 4:** the literature review section should be organised in the same order that the concepts were introduced: art participation, cognition, emotion, and self-efficacy. As a result, I would have expected a conceptual approach in the section on literature review

**Response:** Per the suggestion of the respected reviewer, we managed to organize the literature review based on the structure of the aim of the study.

**Comment 5**: There were a few spelling mistakes, sentence construction issues, and grammatical errors in this and the other sections.

**Response:** We would like to note that a professional editing service has been used to restore the language accuracy and quality of the manuscript.

**Comment 6:** And why do you call this research "cross-sectional"?

**Response:** Since the study conducted a one-time survey. The study design is a cross-sectional survey design.

**Comment 7**: A logical approach to presenting the findings in such a way that they align with the hypothesis would have facilitated reading and comprehension. To address this, I propose that the findings be restructured.

**Response:** We considered the suggestion and the study's finding constructed based on the order of the hypotheses.

**Comment 8**: I find it difficult to associate nonacademic skills as an agent of academic performance. This statement should be clarified in the final paragraph of the discussion section.

### Response: We intended to state that most of the study investigated the application of visual art on children's academic performance, but our study showed evidence of non-academic outcomes.

**Comment 9**: The conclusion should address the two broad areas of focus of this study in order to be complete.

**Response:** Thank you for the suggestion. The conclusion is revised.

**Reviewer 3 Comments**

**Comment 1**: The authors report internal consistency reliability coefficients for these measures but do not indicate why these are needed or their usefulness.

**Response:** Thank you for the suggestions and comments; we learned that it was unnecessary.

**Comment 2:** The dataset is sufficiently large to conduct factor analysis to check the independence and utility of these constructs and provide some psychometric properties and statistical validation.

Response: We appreciate the reviewer for pointing out the essential suggestion. Kindly refer to Table 2 and the finding section for the factor analysis to test whether the data fit a hypothesized measurement model.

**Comment 3:** This paper reads more like a mini-thesis, not an article intended for publication in a journal.

**Response:** We conducted an intensive organization of literature review per the request of the reviewers’ suggestion.

**Comment 4:** The Participants section includes the following statement: “… 2139 students (NFemale = 1182) in grades 9 (MeanAge = 14.32, range = 13-17, SD = 0.561) participating…” Why report the female but not the male participants?

**Response**: As long as the total number of participants is mentioned, it is obvious to know that participants after the female are male participants.

**Comment 5:** Why is it “exciting” that mother’s educational level cannot predict art participation?

**Response:** We have mentioned in the literature and discussion section that in the Chinese context Mother's education level has a strong determiner of the child's quality of life compared to the father's economic status.

**Reviewer 4 Comments**

**Comment 1:** Refer to the PLOS One journal submission guidelines for referencing and other journal reporting formats.

### Response: Suggestion has been taken and we used the journal reference style.

**Comment 2:** It appears the introduction and literature review are not in order. Some ideas are repeated several times in different paragraphs

**Response:** The suggestion has been considered; we rearranged and organized the flow of the manuscript.

.
**Comment 3:** Additional professional editing is needed to improve the manuscript

**Response:** Suggestion has been taken. A professional editing service is used.

**Comment 4:** A reliability test alone is not enough to fully validate a new instrument (or a construct). Other necessary validation analyses, such as EFA and CFA, may be needed to address the various aspects of validity.

**Response:** Suggestions have been considered. Kindly check Table 2 and the CFA analysis report in the result section.

**Comment 5**: The language medium of the instruments is unknown, as is the duration of the data collection. It would be more appreciated if each section in the methodology addressed specific issue(s). For example, issues regarding ethical consideration are scattered. Ethical procedures should also be named appropriately.

**Response:** We addressed such important information per the reviewer's suggestion.

**Comment 6:** Your strict inference that Chinese parents are responsible for what is observed should be considered (under implications, second line). It could be due to several factors.

**Response**: The suggestion is considered and addressed.
