# Supplementary material for: The impact of Chinese adolescents visual art participation on self-efficacy: A serial mediating role of cognition and emotion
Source: PLoS One. 2023 Nov 22;18(11):e0288379. doi: 10.1371/journal.pone.0288379 (PMC10664882; doi:10.1371/journal.pone.0288379)
Supplement: Attachment — Submitted filename: cover letter SR(1)(1).docx [file pone.0288379.s004.docx]

**Cover Letter**

June 23, 2023

Dear Editors and reviewers,

# I am writing this letter on behalf of my coauthor regarding our manuscript Ms. No. PONE-D-23-06961R1, entitled "*The Impact of Chinese Adolescents Visual Art Participation on Self-Efficacy: A Serial Mediating Role of Cognition and Emotion*."We want to express our appreciation to the respected editors and reviewers for providing us the constructive comments and suggestions to shape our manuscript for quality publication. According to our original reviewer's last review, we have obtained critical and rational minor suggestions to complete the manuscript's structure and content before the official publication. As you can see below, we have responded to each comment given by the reviewer corresponding to each page's revision has been made.

**Reviewer 3 Comments**

**Comment 1:** It is understood and expected that a literature review is part of the introduction. The heading "Literature review" should be removed, and a proper transition that leads to the heading "Art participation…" should be inserted.

**Response:** . The suggestion has been considered, and the changes have been made as per the advice 'Art Education' added the heading instead of literature review.

**Comment 2:** Again, stating the number of males and females in the sample is customary and appropriate. Stating one but not the other expects the reader to figure it out! Please state the number of both sexes.

**Response:** Suggestion is considered; we added both sexes number 'with a total of 2139 students (*N_Female_* = 1182, *N_male_* = 957) in grades 9 (*Mean_Age_* = 14.32, range = 13-17, *SD* = 0.561) participating in the study (see Table 1).'

**Comment 3:**When data analysis leads to "exciting" results, the reasons for such excitement should be explicitly stated. The authors highlighted the following sentences to explain why the results are exciting: "which partially negates the second hypothesis," which supports the third hypothesis," and "which validate their (it should be the) fourth hypothesis of this study." I do not believe this is informative, and again, it burdens the reader to figure out why the authors think these results are wonderful. We should always keep the readers in mind and write for them.

**Response:** Thank you for the suggestion; we elaborated on our findings, and in the decision section, we added more details. 'Although a prior Chinese study stated that a mother's education and income have a stronger association with children cultural capital than the father, the present study couldn't support the argument that the target region of this study SES was moderate.'

**Comment 4:** While Table 2 provides the confirmatory factor analysis results, the very few words at the end of the Data analysis procedures are hardly sufficient to address the validity of these scales. Moreover, only the fact that confirmatory factor analysis is performed to validate the hypothesized scales should be stated under the data analysis procedure. The factor analysis results should be part of the results section under its own subheading after descriptive statistics. This part should be thoroughly presented in relation to the validation of the scales. That is, explain the results found in Table 2 and reference them to Table 2.

**Response:** Kindly refer the revised manuscript

**Comment 5:** I do not believe stating chi-squared to have a highly significant value without properly stating the parameter's value is consistent with PLOS style.

**Response:** Suggestion is considered, and changes have made.

**Reviewer 4 Comments**

**Comment 1**: Please consider this sentence. It seems not to be clear. Consider ensuring the free flow of understanding in that paragraph.
1b, "Furthermore, it must be stressed that besides the cognitive development of children through art education, also it strengthens the non-cognitive abilities (self-esteem, self-concept, self-efficacy,peer-relationship, emotions)," (Please replace "also it" with "art education also". Readers are not familiar with the work like you. Please assist them to easily understand your perspective throughout the paper.
2.The use of conjunctive adverbs such as "thus", "in the light of," etc. should be used appropriately (its usage in many places were inappropriate). Example its usage in "Thus, the current study sought to shed light on........ following the sentence "Although children's art participation is significantly dependent....."
Social class implies social groups, ranks, or levels, and so I doubt if "social class level" is appropriate.It appears you are relying solely on AI to deal with syntax and other grammatical issues raised in the previous review. The paper is still not devoid of syntax and other grammatical issues; please seek professional assistance to improve the manuscript or more work need to be done.

**Response:.** The suggestion is considered.

**Comment 2**: The research was conducted in 2022. Data collection spans what period?.

# Response: .Thank you for the reminder, we mentioned the time spans 'sectional survey study's data was conducted in 2022 (between March – May) on junior secondary students from the rural province of Guizhou in southwestern China.'

**Comment 3:** Why is it hard to have a specific section in the methodology that addresses how ethical procedures were properly taken and adhered to in a study involving children? PlosOne requires detailed information on it.

**Response:** . The suggestion is considered; under the methodology, we added a section with 'Ethical Consideration', which provides detailed information.

**Comment 4:** With regard to establishing various aspects of validity, Cite to support your values. For instance, GFI values greater than 0.95 are generally recommended. In your case, we have 0.916, 0.938, 0.942, and 0.885. (It appears something is wrong somewhere.) Hu and Bentler, and others recommend SRMR values be between 0 to 0.08. Three of your figures were 0.113, 0.257, and 0.190. There appears to be a problem with this fit index too. (Perhaps, you can find literature to support this figures.)
You did not also establish discriminant validity or show us the loading for each item. Until these fundamental issues are resolved, it is hard to comment on the rigorousness of the analysis of a study using latent variables. You can see a statistician for support.

**Response:** The suggestion is considered,
